# Simultaneous optic and vestibulocochlear syphilitic neuropathy in a patient with HIV infection

**DOI:** 10.1186/1869-5760-3-27

**Published:** 2013-01-29

**Authors:** Ignacio Rodríguez-Uña, Mercedes Serrador-García, Enrique Santos-Bueso, David Díaz-Valle, Julián García-Feijóo

**Affiliations:** 1 Department of Ophthalmology, Hospital Clínico San Carlos, Instituto de Investigación Sanitaria del Hospital Clínico San Carlos (IdISSC), Complutense University of Madrid, Prof. Martin Lagos Av. S/N, Madrid 28040, Spain

**Keywords:** Hearing loss, HIV infection, Optic neuritis, Syphilis

## Abstract

**Background:**

The purpose of this report is to present a case of optic and vestibulocochlear neuropathy as a manifestation of concurrent HIV and syphilis coinfection. This is an interventional case report of a 37-year-old man who complained of blurry central vision in his left eye and hearing loss in his left ear over the past 2 weeks.

**Findings:**

Visual acuity was 20/20 in both eyes, and the anterior segment was normal in both eyes without relative afferent pupillary defect. Fundoscopy revealed swelling of the left optic disc. Optic coherence tomography and the Heidelberg retina tomograph confirmed and quantified the oedema of the left optic disc. An audiometry showed a left sensorineural deafness. Serological examinations disclosed confirmed HIV and syphilis infection. Magnetic resonance imaging of the brain showed no abnormalities. Properly treated with intravenous penicillin, the lesions resolved.

**Conclusions:**

Simultaneous optic and auditive involvement can be the first manifestation of syphilitic and HIV coinfection. To our knowledge, this report is the first to describe the rare occurrence of syphilitic optic neuritis and ipsilateral affectation of the vestibulocochlear nerve.

## Findings

### Introduction

The incidence of syphilis continues to rise in the USA and Europe, and it is estimated that around 20% of patients with syphilis in the USA also have HIV infection [1-3]. Syphilis may present as multiple clinical entities, and the ocular manifestations of syphilis may involve any structure of the eye [3]. The observation of optic nerve abnormalities in an ophthalmological examination in a patient with syphilis is highly suggestive of central nervous system (CNS) involvement and should be considered synonymous with neurosyphilis [4,5]. Syphilis is also a well-known cause of hearing loss [6]. In a MEDLINE search of the literature, we were unable to find previous reports of optic and vestibulocochlear neuropathy as simultaneous manifestations of syphilis and HIV coinfection.

## Case report

**Figure 1:**
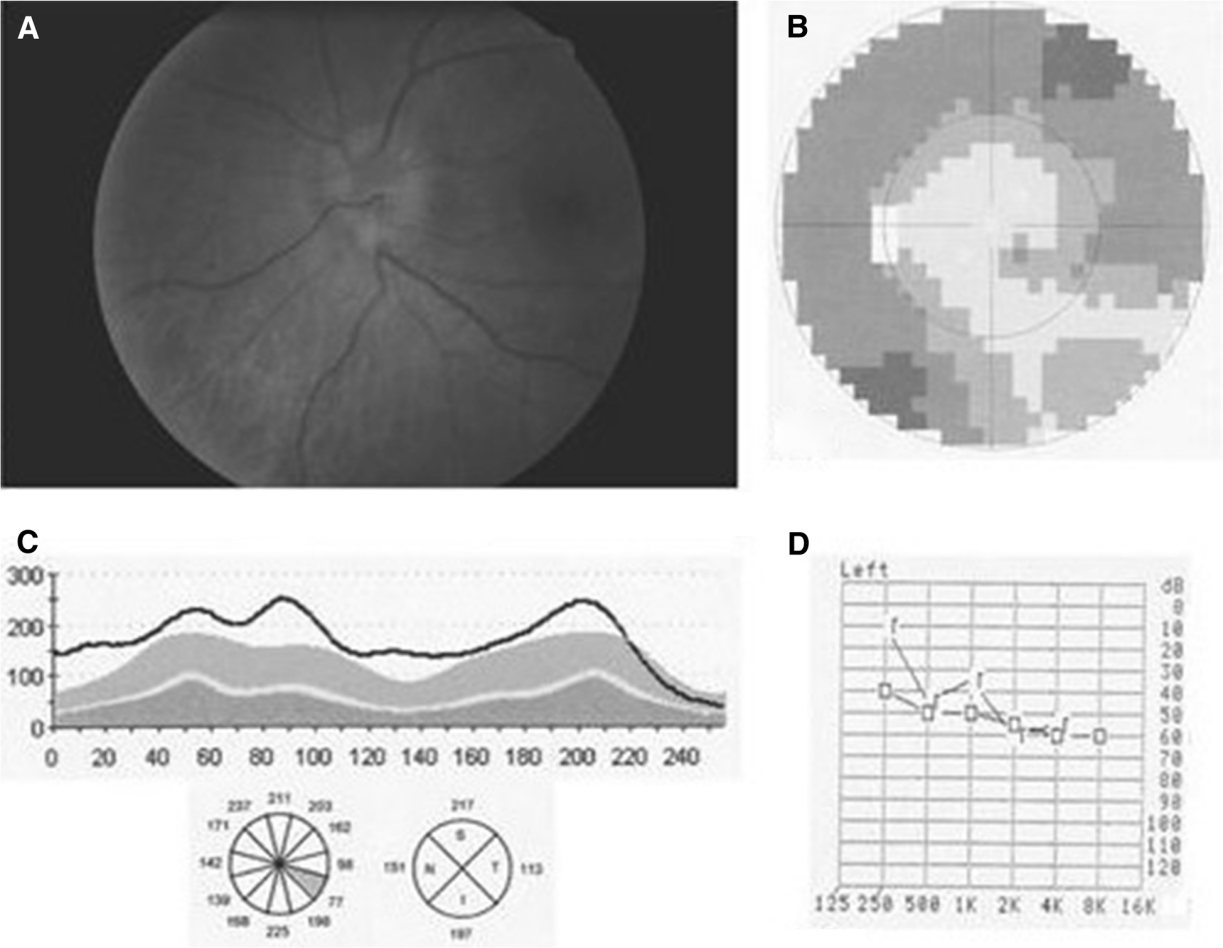
**Left eye at presentation. **(**A**) Fundoscopy reveals swelling of the left optic disc. (**B**) Reduced peripheral sensitivity detected in a visual field test. (**C**) OCT quantifying the oedema in the left optic disc. Note the RNFL thickening in all quadrants and in 11 of 12 RNFL sectors. (**D**) Audiometry of the left ear indicating impaired sound transmission both through the air and bone. The greater effects observed for the higher frequencies are diagnostic of sensorineural deafness.

A 37-year-old man complained of blurry central vision in his left eye (LE), hearing loss in his left ear and recent fatigue over the past 2 weeks. In a general examination, a macular skin rash was detected. Visual acuity (VA) was 20/20 in both eyes. An anterior segment examination was normal in both eyes with no relative afferent pupillary defect. Intraocular pressure was 12 mmHg in both eyes. Fundoscopy revealed swelling of the left optic disc (Figure 1A), with no abnormalities detected in the right eye (RE). A visual field test proved normal for the RE, while the LE showed reduced peripheral sensitivity (Figure 1B). Optical coherence tomography (OCT; Spectralis (new model), Heidelberg Engineering, Heidelberg, Germany) and laser confocal microscopy (HRT-III; Heidelberg retina tomograph, Heidelberg Engineering, Heidelberg, Germany) confirmed and quantified the oedema in the left optic disc. OCT of the LE revealed considerable retinal nerve fibre layer (RNFL) thickening in all quadrants and in 11 of 12 RNFL sectors (Figure 1C). OCT of the RE was unremarkable.

The results of the rapid plasma reagin (RPR) test were positive for syphilis (titre 1:64). The findings of a lumbar puncture were as follows: RPR was negative, fluorescent treponemal antibody absorption test was negative and no immunoglobulin G antibodies to *Treponema pallidum* were detected. Blood results indicated an HIV-1 RNA polymerase chain reaction of 80,990 copies/ml and CD4 T cell count of 542 cells/μl, taken as positive for HIV infection. No abnormalities were detected during magnetic resonance imaging of the brain. Audiometry of the left ear revealed impaired sound transmission both through the air and bone, with greater effects observed for the higher frequencies (Figure 1D), and a diagnosis of sensorineural deafness was made. The audiometric test result for the right ear was normal.

**Figure 2:**
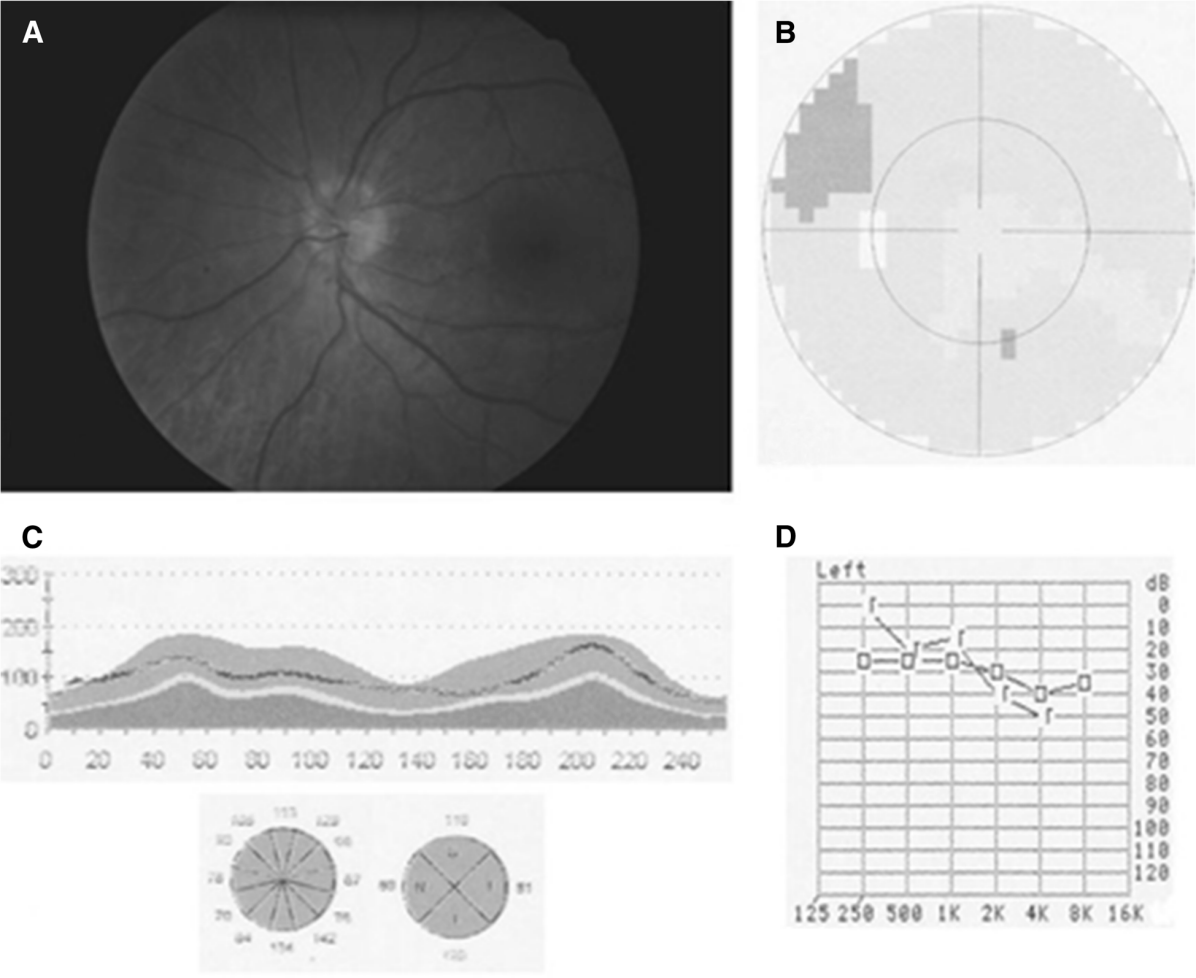
**Three weeks after treatment with intravenous penicillin. **(**A**) Fundoscopy reveals the absence of oedema in the left optic disc. (**B**) Left eye visual field test is normal. (**C**) Normal OCT-determined RNFL thickness indicates resolution of the oedema. (**D**) Audiometric test results for the left ear are normal.

A diagnosis of luetic optic and ear neuritis with concurrent HIV infection was made, and the patient was treated with intravenous penicillin (four million units given every 4 h for 2 weeks) and with intramuscular penicillin (2.4 million units/week for 3 weeks) simultaneously. Treatment was able to resolve the visual and hearing disturbances. The patient was not started on antiretroviral therapy because his CD4 T cell count was still over 350 cells/μl. Three weeks after initiation of treatment and 3 months after the onset of symptoms, fundoscopy revealed the absence of oedema in the left optic disc (Figure 2A), and VA was 20/20 in both eyes. Perimetry was normal (Figure 2B), and OCT-determined RNFL thickness was within the limits of normality (Figure 2C). The patient reported a marked improvement of hearing in his left ear, which was confirmed by audiometry (Figure 2D).

## Discussion

CNS involvement can occur at any stage of syphilis. Among patients with secondary syphilis, around 18% may have neurological signs (including ocular disease) or symptoms [7]. Ocular involvement may be silent or present as anterior uveitis, choroiditis, interstitial keratitis, retinal vasculitis, retinitis, optic neuritis, dacryoadenitis or scleritis. Tamesis and Foster reported that uveitis was the most common ocular manifestation in ocular syphilis [8]. Ocular syphilis has been reported in both immunocompromised and immunocompetent individuals [1]. Due to the wide variety of clinical forms it can take, syphilis has been referred to as ‘the great pretender or simulator’ and should be ruled out in all patients with ocular inflammation. Optic nerve involvement in syphilis may be unilateral or bilateral and becomes apparent as perineuritis, anterior or retrobulbar optic neuritis or papilloedema. In the present case, a unilateral optic disc oedema was detected, which disappeared slowly until its final resolution 3 months after the onset of symptoms. Beyond this optic disc oedema, an area of optic atrophy was observed by OCT. However, the patient's visual field was unaffected, and no changes indicative of recovery were detected. Thus, in the present case, OCT (to quantify optic disc oedema) was more sensitive than perimetry at identifying and monitoring optic neuritis [9].

Posterior segment and optic nerve involvement can be an important manifestation of ocular syphilis associated to HIV infection [10], and as in our patient, it often leads to the initial HIV diagnosis [11]. In this case, the HIV infection was coincidental and was investigated due to the diagnosis of syphilis. Moreover, a patient with a first-discovered HIV infection should also be checked for syphilis. A higher incidence of neurosyphilis occurs in syphilis concurrent with HIV, may give rise to ocular complications and may show a more aggressive course [12,13]. In addition, acquired syphilis, both secondary and tertiary, may cause deafness, which is usually unilateral, although in some patients can be bilateral [6].

For some time, the treatment of choice for neurosyphilis has been prolonged high-dose intravenous penicillin and has been related to a good prognosis for visual and hearing impairment [13]. The successful use of ceftriaxone has also been described [9]. Oral and intravenous corticosteroids are commonly given as adjuvants for posterior uveitis, scleritis and optic neuritis. Penicillin plus corticosteroid-based regimens have also proved effective at improving hearing, tinnitus and vertigo. The factors associated with a good response include fluctuating symptoms, especially hearing, hearing loss with a duration of less than 5 years and an age under 60 years [14].

This report describes an infrequent case of unilateral optic neuropathy and ipsilateral involvement of the vestibulocochlear nerve as simultaneously presenting symptoms of syphilis and HIV coinfection.

## Competing interests

The authors declare that they have no competing interests.

## Authors’ contributions

IR performed the bibliographic search, developed the study and drafted the manuscript. MS participated in the design of the study. ES conceived the study and participated in its coordination. DD developed the study, participated in its coordination and in the bibliographic review. JG participated in the coordination of the study. All authors read and approved the final manuscript.

## Consent section

Written informed consent was obtained from the patient for publication of this report and any accompanying images. This work was performed with the approval of the Ethics Committee of the Hospital Clínico San Carlos in Madrid (Spain).
